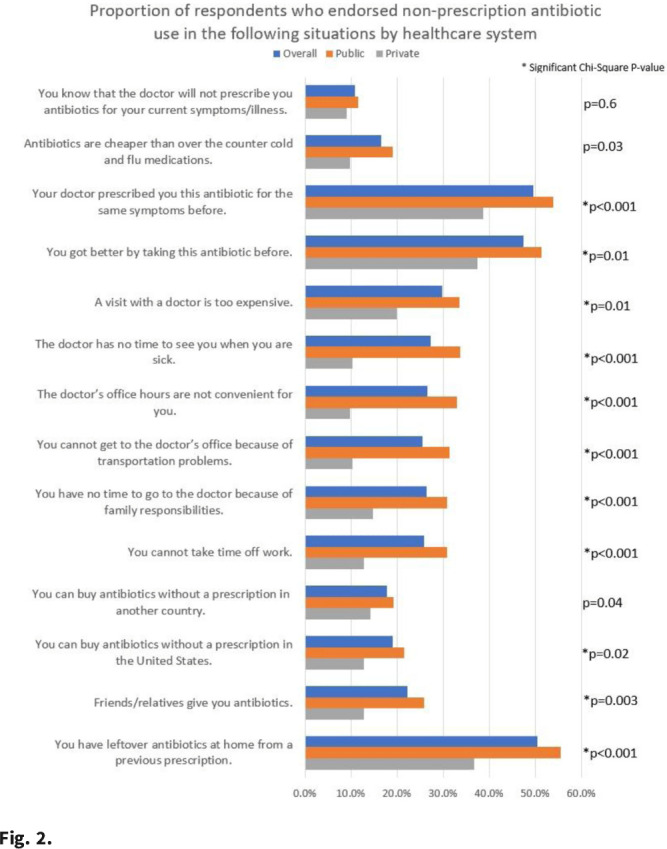# Identifying symptoms/illnesses and situations that predispose outpatients to use antibiotics in two healthcare systems

**DOI:** 10.1017/ash.2022.189

**Published:** 2022-05-16

**Authors:** Lindsey Laytner, Larissa Grigoryan, Barbara Trautner, Osvaldo Alquicira, Juanita Salinas, Michael Hansen, Roger Zoorob, Fareed M. Khan

## Abstract

**Background:** Taking antibiotics outside the guidance of a clinician (nonprescription use) is a potential safety issue and runs counter to antibiotic stewardship efforts. We identified the symptoms and illnesses and situations that may predispose patients to take antibiotics, and we compared these findings between patients attending public primary care clinics and private emergency departments. **Methods:** A cross-sectional survey was conducted between January 2020 and March 2021 in 6 primary care clinics and 2 emergency departments in the United States. We queried patients about 5 symptoms and illnesses (Fig. 1) and 14 situations (Fig. 2) to investigate whether these would lead the patients to take antibiotics without a prescription. We used the χ^2^ test to compare the symptoms and illnesses and situations between the respondents from public and private healthcare systems. We set the *P* value for significance at <.025. **Results:** In total, the survey had 564 respondents (median age, 49.7 years; range, 19–92), and 72% were female. Most respondents identified as either Hispanic or Latina/Latino (46.6%) or African American or Black (33%), followed by White (15.8%), and other (4.6%). Most respondents had visited public clinics (72%). The most common insurance status for our respondents included Medicaid or county financial assistance program (56.6%), followed by private insurance or Medicare (36.7%) and self-pay (6.7%). In public primary care clinics, only 23% had private insurance or Medicare compared to 72.9% in private emergency departments. Of those surveyed, 69% agreed that antibiotics would improve the recovery from sinus infections, followed by bronchitis (64%), sore throat (64%), cold/flu (61.4%), and diarrhea (31.5%). The proportions of respondents who believed that antibiotics would improve the recovery from diarrhea (36.2% vs 19.4%; *P* = .004) and sore throat (59.9% vs 48.4%; *P* < .001) were significantly higher among public versus private outpatient respondents. We did not find significant differences for cold/flu, sinus infection, or bronchitis between these 2 healthcare systems (Fig. 1). In 11 of the 14 situations, patients in public clinics were more likely to report a likelihood of using nonprescription antibiotics than the patients visiting the private emergency rooms (Fig. 2). **Conclusions:** Future stewardship interventions should be aware of the symptoms and illnesses and situations that may influence outpatients to take nonprescription antibiotics. Addressing modifiable factors (eg, leftover antibiotics, antibiotics given by friends or family, and antibiotics available without a prescription in stores or markets) may also curtail these unsafe practices and reduce antibiotic resistance.

**Funding:** None

**Disclosures:** None

**Figure:**
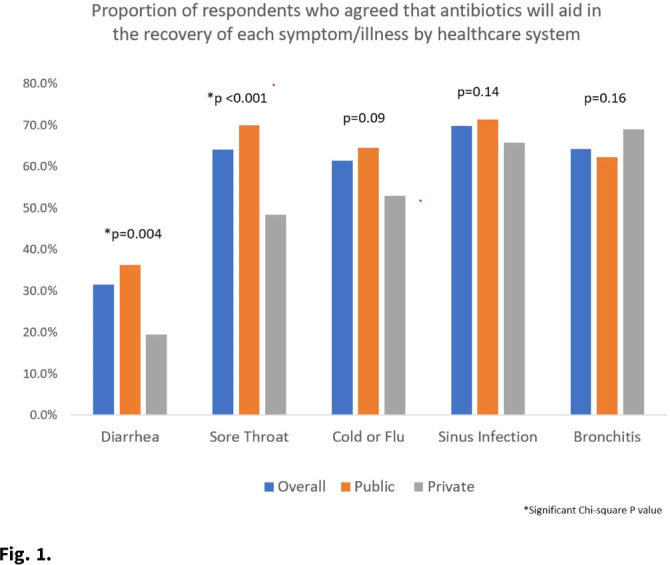

**Figure:**