# Identifying symptoms/illnesses and situations that predispose outpatients to use antibiotics in two healthcare systems – CORRIGENDUM

**DOI:** 10.1017/ash.2022.296

**Published:** 2022-10-04

**Authors:** Lindsey Laytner, Barbara Trautner, Michael Hansen, Roger Zoorob, Osvaldo Alquicira, Juanita Salinas, Fareed M. Khan, Larissa Grigoryan

In the above abstract^1^, the listing of the author names should read as follows, according to seniority:

Lindsey Laytner, Barbara Trautner, Michael Hansen, Roger Zoorob, Osvaldo Alquicira, Juanita Salinas, Fareed M. Khan and Larissa Grigoryan
